# Mutations in normal tissues—some diagnostic and clinical implications

**DOI:** 10.1186/s12916-020-01763-y

**Published:** 2020-10-29

**Authors:** Clare Fiala, Eleftherios P. Diamandis

**Affiliations:** 1 Department of Pathology and Laboratory Medicine, Mount Sinai Hospital, Toronto, ON Canada; 2 Department of Laboratory Medicine and Pathobiology, University of Toronto, Toronto, ON Canada; 3 Department of Clinical Biochemistry, Mount Sinai Hospital and University Health Network, 60 Murray St. Box 32, Floor 6, Rm L6-201, Toronto, ON MST 3L9 Canada

**Keywords:** Normal tissues, Mutations, Circulating tumor DNA, Driver mutations, Passenger mutations

## Abstract

**Background:**

It has long been known that mutations are at the core of many diseases, most notably cancer. Mutational analysis of tissues and fluids is useful for cancer and other disease diagnosis and management.

**Main body:**

The prevailing cancer development hypothesis posits that cancer originates from mutations in cancer-driving genes that accumulate in tissues over time. These mutations then confer special characteristics to cancer cells, known as the hallmarks of cancer. Mutations in specific driver genes can lead to the formation of cancerous subclones and mutation risk increases with age. New research has revealed an unexpectedly large number of mutations in normal tissues; these findings could have significant implications to the understanding of the pathobiology of cancer and for disease diagnosis and therapy. Here, we discuss how the prevalence of mutations in normal tissues provides novel and relevant insights about clonal development in cancer and other diseases. Specifically, this review will focus on discussing mutations in normal tissues in the context of developing specific, circulating tumor DNA (ctDNA) tests for cancer, and evaluating clonal hematopoiesis as a predictor of blood cancers and cardiovascular pathology, as well as their implications to the phenomena of neural mosaicism in the context of Alzheimer’s disease.

**Conclusions:**

In view of these new findings, the fundamental differences between the accumulation of genetic alterations in healthy, aging tissues compared to cancer and cardiovascular or neural diseases will need to be better delineated in the future.

## Background

In 1990, Fearon and Vogelstein presented a hypothesis, whereby cancer development is the result of accumulating mutations in some critical genes, occurring over years [1]. Hanahan and Weinberg provided more granularity to this model by identifying eight hallmarks of cancer that are a consequence of genetic instability [2]. However, very recent and continually expanding data suggest that normal tissues, and particularly aging tissues, accumulate a surprisingly large number of mutations that are frequently inconsequential, prompting some to speculate that this is the “new normal.” Several studies have found an approximate range of human germline mutation rates: 1.0–1.2 × 10^−8^ per nucleotide per generation and up to 10% of these mutations are deleterious to some degree [3, 4].

The search to delineate the pathway of tissue mutations from harmless to pathological marks a new frontier for cellular and molecular research—beyond simply detecting and classifying genetic variations but determining whether these mutations will lead to disease. However, this challenge is complicated by various exogenous and endogenous factors. The transition from a benign clone to tumorigenesis or the accumulation of genetic mutations that predispose an individual to Alzheimer’s or cardiovascular disease is intimately influenced by the patient’s immune system, the environment, other comorbidities, and related epigenetic changes. For example, smoking is well known to augment mutagenesis in lung tissues [5], and cirrhosis (often caused by alcoholism or viral hepatitis) can also lead to increased driver mutations and clonal expansion in liver tissue [6]. Even water and oxygen, fundamentally necessary for life, can significantly mutate DNA. However, the percentage of oncogenic mutations in cells of normal tissues may be quite variable and may need highly sensitive assays in order to be detected or excluded. Clearly, more experimentation is necessary in order to make more robust predictions.

**Fig. 1:**
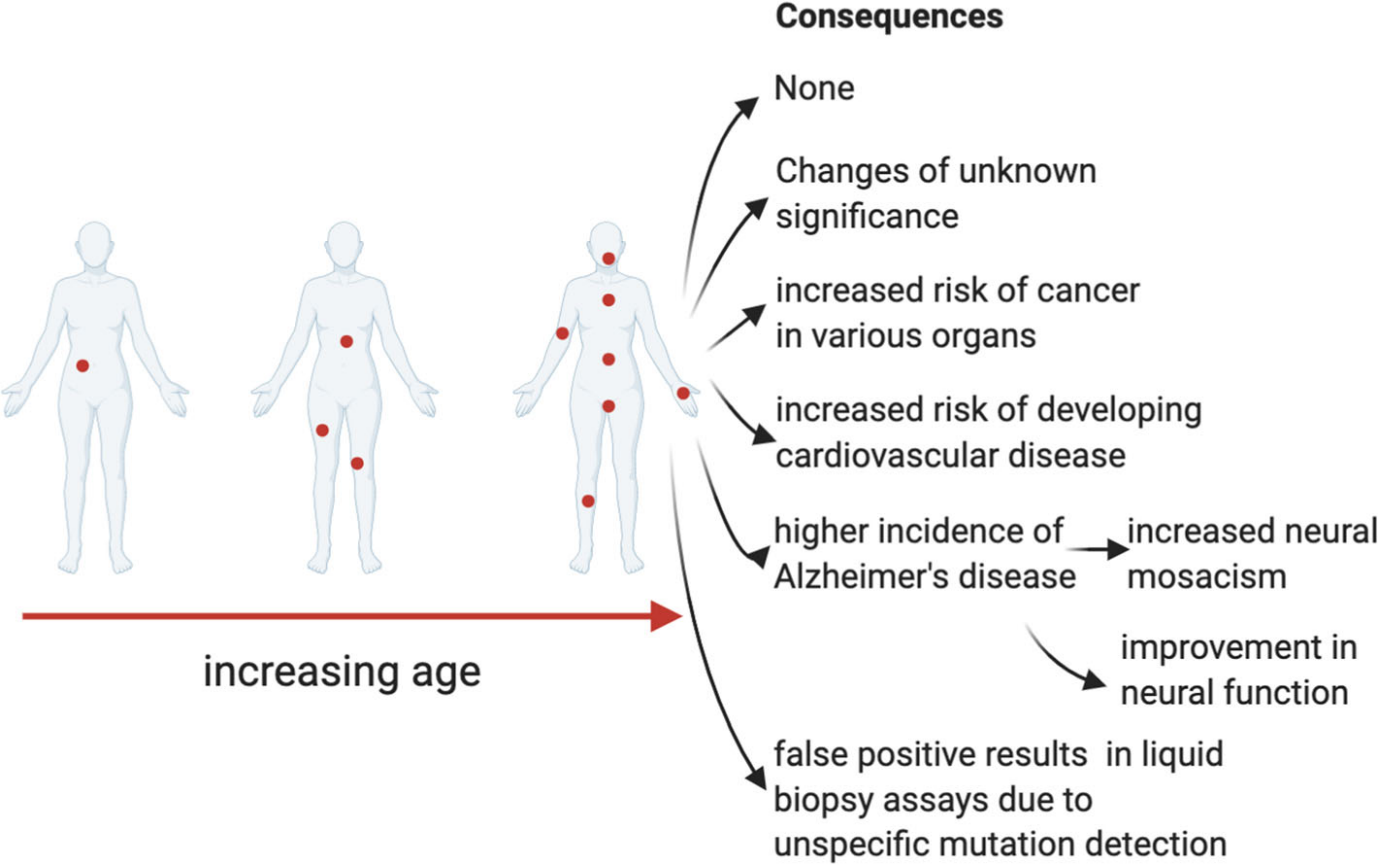
Potential consequences of accumulating genomic changes with age (including mutations) in normal tissues. Figure created using BioRender

Further investigation into the mechanisms underlying mutations in normal tissues will likely uncover new opportunities for early detection as well as an increased understanding and even development of reference intervals to delineate which genetic variations are pathological and which are not. A visual summary of the potential consequences of the age-related accumulation of genomic changes in normal tissues can be found in Fig. 1.

## Clonal development, circulating tumor DNA, and cancer detection

Biopsies are a cornerstone for cancer treatment, and in the last decade, tumor genetic sequencing has been extensively used to guide treatment selection and prognosis determination.

The inherent invasiveness of surgical resection and biopsy means these procedures are performed relatively infrequently. Previously, genome sequencing technologies have been focused on determining cancer-related mutations from patients diagnosed clinically or through screening. However, now, there is great interest in turning these technologies and knowledge towards early detection which necessitates knowing exactly which mutations signify cancer and which mutations constitute benign passenger changes.

Cells are thought to release their DNA into the circulation through apoptosis and necrosis; this genetic material is known as circulating free DNA (cfDNA). For example, during pregnancy, some fetal cells and fetal DNA escape into the maternal circulation which can be used to test the fetus for genetic abnormalities. Cancer cells also release DNA, denoted circulating tumor DNA (ctDNA). New tests (known widely as liquid biopsies) involve extracting and sequencing this ctDNA from a blood sample, a highly desirable alternative to tissue biopsies. Not only are liquid biopsies minimally invasive, ctDNA tests can be repeated frequently to analyze cancer progression or regression throughout treatment. Many investigators have used this technique to monitor cancer burden, treatment response, and tumor evolution in patients already diagnosed with the disease. The US Food and Drug Administration (FDA) has already approved a ctDNA test involving monitoring EGFR (epidermal growth factor receptor) mutations in the serum of non-small cell lung cancer patients to determine eligibility for the chemotherapy drug erlotinib [7], and another test to determine ctDNA residue after surgery has been granted breakthrough device status [8].

However, now, investigators are endeavoring to develop a blood test for early detection of cancer in healthy individuals without symptoms or tumors invisible on imaging. The tests would involve detecting and sequencing the tiny amounts of ctDNA from the tumors that could be less than 2 mm in diameter (4 mm is the approximate detection limit of imaging for tumor detection) [9]. There are several challenges involved in developing such a test, including the high possibility of sampling error due to low mutant allele fraction caused by the miniscule tumor volume [10]. Moreover, there are also methodological difficulties involved with detecting somatic mutations in normal tissues that may only occur in small cell populations. Accuracy is crucial because false detection of cancer can lead to unnecessary procedures and surgery as well as significant patient anxiety and cost to the health care system. The concerns about specificity are most relevant to this review. A ctDNA test’s success for early diagnosis is predicated on determining which mutations indicate cancer and which are associated with benign diseases, a task that is becoming more complicated as new research suggests that significant mutagenicity in normal tissues is the norm, not the exception.

### Pre-cancerous clonal development

A recent study by Yizhak et al. examining more than 6700 histologically healthy samples spanning 29 types of tissues from almost 500 individuals underscores this phenomenon [11]. Employing analyses of RNA sequence databases to identify somatic mutations, the team found many mutations generally associated with cancer as well as macroscopic clones (population of mutated cells descended from a common ancestor, visible without a microscope) in tissues without any cancer pathology. Specifically, 95% of the study participants had at least one tissue with macroscopic clonal cell populations, 40% had one large mutational clone, and 5% of tissues had 5 or more large mutational clones. Moreover, 33% of the healthy participants carried a gene mutation connected to cancer. Unsurprisingly, the number of these mutations increased with age and with cell proliferation rates; high cell turnover tissues such as the skin and esophagus had increased levels of mutations compared to the less proliferative brain and muscle cells [11]. As expected, the tissues most affected by endogenous factors such as sun exposure and smoking—skin and lung—had a higher mutational burden, reinforcing the significant connection between environmental factors and mutagenicity.

The specificity of ctDNA analysis for early cancer diagnosis is complicated by the phenomenon of mosaicism: age-related genetic mutations occurring in a subset of an individual’s cells. Consequently, even if a sensitive assay is developed to reliably detect mutant cfDNA at low concentrations in the circulation, investigators must be able to consistently determine whether the mutations are cause for concern. Sun-exposed human skin is the most highly mutated tissue—a 2015 study found driver mutations in about 25% of skin cells from healthy participants [12]. Another study employed mathematical modeling of tumor development and determined that more than half of the mutations found in some cancers were present in the normal cell where the cancer began—the founder clone. Thus, these somatic mutations found would have been present even if the tumor had not formed [13].

A recent study of more than 2000 colonic crypts further illustrates this challenge [14]. The authors found that approximately 1% of normal colorectal crypts in middle age individuals contain a driver mutation, yet incidence of colorectal cancer is much lower, suggesting that these tumors are rare incidences of normal mutational processes in aging colorectal epithelium. These discoveries dovetail with clinical finding from other investigations. Only 5% of people develop colorectal cancer across their lifespan [15] even though adenomas are found via routine colonoscopy in approximately 40% of individuals over 70 years old [16]. In fact, there is a less than 1 in 375,000 chance that the crypt micro neoplasms detected in the previous study would become macroscopically detectable adenomas and less than 1 in 3 million chance that they become carcinomas [14].

### Mutations in esophageal carcinoma

Other research with esophageal squamous cell carcinoma has further elucidated the underlying process influencing whether a mutation will lead to the formation of cancerous clones. Martincorena et al. analyzed 844 upper esophageal tissue samples from 9 healthy donors (aged 20 to 75 years) [17]. Most importantly, the team discovered that cancer-associated genes were mutated far more frequently in healthy esophagus samples than in healthy skin. In fact, many of these mutated genes were under strong positive selection, leading to increased cell proliferation and the consequent formation of cell clones in the esophagus. Unsurprisingly, there was an increased number of mutations in both overall and in driver genes as well as a larger clone size on average in the tissue samples from older participants compared to younger participants. Another important finding was though mutations connected to benign clonal expansion may appear frequently in cancer cell genomes, these particular mutations may not necessarily contribute to carcinogenesis because these tissues already have a naturally high mutation frequency. Furthermore, an analysis by Higa and DeGregori demonstrated that even classic “oncogenic drivers” like *NOTCH1* might not often contribute to carcinogenesis [18].

Even though the esophagus does not receive the same mutagenic exposure to ultraviolet radiation compared to skin and has a tenth of the mutations compared to skin, it is still exposed to a significant number of mutagens through a human’s varied diet [19]. Consequently, Martinconera et al. found that some genes were frequently mutated in both skin and esophageal tissue, particularly *NOTCH1*, *TP53*, *NOTCH2*, *FAT1*, *NOTCH3*, and *ARID1A* [17]. Most of the abnormalities were missense and protein-truncating mutations though CpG islands were also heavily mutated [17].

Previous investigations reported mutations in *TP53* in more than 90% of esophageal squamous cell carcinoma (OSCC) cases [20]. Conversely, Martincorena et al. found *NOTCH1*to be the most commonly mutated gene in healthy esophageal tissue while *TP53* mutations were far less prevalent in their (healthy) samples [17]. Even though Marincorena’s team detected a significant number of mutations in cancer-associated genes, their participants’ tissues still appeared healthy. Notably, the rate of incidence of esophageal cancer is less than 0.01% of the general population [21], far less than the incidence suggested by the number of *NOTCH1*and *TP53*mutations observed by Marincorena et al. in healthy tissues [21].

Significantly, these results were echoed in a similar study by Yokoyama et al. which used both normal and cancerous esophageal tissue samples [22]. Age-related mutational signatures were most prevalent in the healthy samples whereas mutational signatures associated with the cytidine deaminase *APOBEC* (an mRNA modifying enzyme) or alcohol were most commonly found in cancer samples [22]. The studies with esophageal tissue provide compelling new information about the age-related accumulation of mutations and their transition to cancer. They also show the value of determining which specific gene mutations or mutational signatures most often lead to particular malignancies, crucial information for developing accurate ctDNA liquid biopsies for diagnosis as well as other related genetic tests.

### Mutations in the female reproductive system

A study with paired peritoneal fluid and blood samples from women without cancer detected mutations in *TP53* at low frequencies (< 0.01%) in 16 out the 17 participants [23]. Moreover, the non-synonymous mutations were enriched, undergoing positive selection. Another investigation focused on endometriosis, a painful though benign pathology of the female reproductive system which rarely leads to cancer [24]. Yet, researchers found mutations in some important proto-oncogene driver genes with a high mutant allele fraction including *KRAS*, *PIK3CA*, *PTEN*, *ARID1A*, and*TP53* in 19 out 24 patients’ endometriotic lesions [24]. Notably, these women had no sign of neoplasia; however, some samples had mutant allele fraction (MAF) as high as 40%, showing the extensive prevalence of these mutations [24]. A similar study with uterine lavage fluid samples from healthy women echoed these results with more than half of 95 healthy women having cancerous driver mutations, positively correlating mutations in cancer-causing genes with age and postmenopausal status [25]. Finally, a recent study by Moore et al. on putative oncogenic driver mutations in normal endometrium found that its mutational burden increased at approximately 29 base substitutions each year [26]. Significantly, the particular mutational composition of the epithelium varied widely among the 28 women studied.

### Mutations in other solid tissues

*TP53* mutations have been reported in histologically normal oral, bronchial, bladder, and esophageal epithelial tissues [27]. *KRAS* mutations have also been detected in normal tissues adjacent to colorectal and lung cancers [28]. As expected, copy number variations are especially abundant in rapidly dividing tissues, particularly with genes involved in growth regulation [29]. Comparable mutations were also discovered in healthy eyelid skin, removed during blepharectomy [12]. Another study examined kidney, fat, and muscle cells from donors of different ages [30]. The investigations identified a mutation-prone cell type in the kidney while also delineating an age-related decline in DNA repair capacity. Similar work on mutational processes has also been conducted in the lung. One important finding was that following smoking cessation cells with a lower mutation burden are replenished since the lung is no longer exposed to mutagenic carcinogens [5].

A fascinating study showed a similar number of mutations in adult stem cells from small intestine, colon, and liver tissues even though there are very different incidences of these cancer in the general population [31]. Liver cancer and colon cancer are approximately 4 and 10 times, respectively, more prevalent in the USA than small intestine cancer [32–34], highlighting that the number of mutations is not necessarily a faultless indicator of cancer and there are likely tissue-dependent and external factors that influence tumor development.

The extensive range of studies of mutations in normal tissues highlights the specificity challenges relevant to ctDNA test development and mutation classification, spanning a range of cancers and tissue types [35]. Moreover, many of the mutations are present in multiple types of cancer, which would make it even more difficult to determine the tumor’s tissue of origin after a positive ctDNA test. A complex net of biochemical, epigenetic, and environmental factors likely influences whether mutations remain harmless or a person develops cancer. Consequently, there is a need for additional studies to delineate these mechanisms, determining which particular genes are more likely to be mutated for specific cancers, and which mutations are simply hallmarks of old age. In the meantime, the presence of mutations in normal tissues remains a major caveat to the development and specificity of a ctDNA test for the diagnosis of cancer.

## Clonal hematopoiesis: hematological malignancies and cardiovascular disease

Clonal hematopoiesis, the formation of a genetically distinct population of blood cells, is another example of mutations in normal tissues. Clonal hematopoiesis is thought to precede many hematologic cancers, including acute myeloid leukemia (AML) and chronic lymphocytic leukemia [36–38]. For example, a recent study of peripheral blood cells obtained from 95 patients a mean of 6.3 years before AML diagnosis found that they had higher variant allele frequencies and greater clonal expansion compared to controls [39]. However, putative driver mutations connected to age-related clonal hematopoiesis (ARCH-PD) were not found exclusively in the pre-AML patients. 36.7% of controls had observable ARCH-PD compared to 73.4% of patients. Thirty-nine percent of patients above age 50 in the pre-AML cohort had a driver mutation with a variant allele frequency greater than 10%, in contrast to only 4% of controls. A landmark study of 12,380 patients elucidated some of the clinical implications of clonal hematopoiesis [40]. The researchers found clonal hematopoiesis increases with age, observing related somatic mutations in 10% of participants older than age 65. However, rates of clonal hematopoiesis dropped to less than 1% for participants younger than 50. *DNMT3A*, *ASXL1*, *TET2*, *PPMD1*, and *JAK2*were determined to be the most commonly mutated genes in the cohort’s blood cells, with the number of mutations increasing with age [40].

Even though clonal hematopoiesis was a strong risk factor for hematologic cancers, with 42% of the hematologic cancers arising in people with clonal hematopoiesis, a significant portion of the cohort identified to have clonal hematopoiesis did not develop cancer over a 2- to7-year follow-up period [40]. These individuals had clonal hematopoiesis of indeterminate potential, referring to expanded blood cell clones in the absence of other abnormalities. As a result, testing for clonal hematopoiesis is not necessarily a sensitive predictor of cancer, and it is not clear what factors precipitate the transition from clonal hematopoiesis to cancer.

A study of neonate cord blood samples found that around 1% had *TEL-AML1* and *AML1-ETO* gene fusions, which have been correlated with increased chance of developing leukemia [41]. However, longitudinal studies with children carrying such mutations would be useful to determine if these mutations actually predict leukemia incidence and what exogenous factors predict disease onset. Another large study involving nested case-control analyses of 4726 participants with heart disease and 3529 controls linked clonal hematopoiesis with a two times greater incidence of atherosclerosis, a condition characterized by arterial buildups of fat and cholesterol [42]. *TET2* is one of the most commonly mutated genes related to clonal hematopoiesis, and mice that received bone marrow grafts from homozygous or heterozygous *TET2* knockout mice developed larger atherosclerotic lesions compared to the control mice [42]. Yet again, not all the study participants with clonal hematopoiesis developed this disease. Further investigation in mice and in vitro models revealed that TET2 deficiency mediated by clonal hematopoiesis accelerated atherosclerosis, a disease mediated by chronic inflammation, by increasing macrophage proliferation [43, 44]. While factors such as diet and exercise likely play an important role, there is still a need to elucidate the biochemical processes underlying why some people with clonal hematopoiesis developed atherosclerosis and others did not. Clonal hematopoiesis has also been linked to greater risk of coronary heart disease, ischemic stroke, and overall mortality; however, more longitudinal studies are required to confirm these preliminary associations [36].

Even though clonal hematopoiesis testing for cancer and cardiac disease is attractive because it is essentially non-invasive, it still has a very long way to go before it reaches the clinic. Clonal hematopoiesis can also complicate chemotherapy as different subsets of cells have slightly different genomes, making them resistant to particular cytotoxic agents. In fact, recent research using blood samples from approximately 50,000 cancer-free individuals found that positive selection for advantageous clonal mutations, not neutral genetic drift, constituted the major influencer on clonal hematopoiesis and clone fitness, providing further insight into the complex role of clonal hematopoiesis and disease development [45, 46]. While the aforementioned studies represent a good start, more work and sequencing methods with greater sensitivity are needed to precisely determine which of the associated mutations or groups of mutations in blood cells are carcinogenic and pathological, or simply hallmarks of aging normal tissues.

## Mosaicism in the brain and Alzheimer’s disease

Increased mosaicism—genetic changes/mutations in a subset of an organism’s cells—has also been found in neurons, both from healthy people and those with Alzheimer’s disease [47]. The exact mechanism is unknown; however, recent research suggests that amyloid-β precursor protein (*APP*) mRNA variants became permanently incorporated into the genome of neurons due to breaks in neuronal DNA and the action of reverse transcriptase. The investigators termed these novel genome sequences genomic complementary DNAs (gencDNAs) and identified them in the neurons of individuals with Alzheimer’s disease as well as in those who were cognitively healthy [47]. Neurons are thought to be more susceptible to the incorporation of gencDNA due to their long lifespans and the fact that they rarely divide. Moreover, this mutational mechanism introduces another risk factor for individuals who suffered traumatic brain injury—the ensuing breaks in DNA could provide opportunities for Alzheimer-related gencDNA formation [47].

As expected, the number of gencDNAs increased with age, and the researchers found 10 times more gencDNAs in neurons from people with Alzheimer’s disease than in cognitively healthy participants [47]. Some of these *APP* mRNA variants are thought to translate into cytotoxic proteins, further underscoring the role of mosaicism in Alzheimer-related neuron death. It is important to note that this is still very preliminary work and the authors were not able to conclude whether the accumulation of gencDNAs is a consequence or a cause of Alzheimer’s disease [47]. Regardless, this phenomenon of DNA shuffling in somatic cells has only ever been reported in the immune system to generate antibody diversity, and its presence in the central nervous system is a fascinating discovery relating mutations found in normal tissues.

In fact, some mutations occurring in normal brain tissue may even be beneficial. The authors speculate that APP gencDNAs may contribute to synaptic proteome diversity, producing different genes that may improve neuron function [47]. Different clusters of neurons may be modified for selective activities, reducing the need for alternative splicing or RNA modification. gencDNAs may also be important for normal brain function, playing a role in plasticity, learning and memory, or giving neurons the ability to remember and employ genetic variants beyond the wild-type genes [47].

The discovery of the increase of gencDNAs in sporadic Alzheimer’s disease and its reliance on reverse transcriptase may even offer a promising therapeutic approach. It is known that people who have human immunodeficiency virus and have been on long-term anti-retroviral therapy including reverse transcriptase inhibitors have a reduced incidence of Alzheimer’s disease compared to the rest of the population [48, 49]. Thus, reverse transcriptase inhibitors may be a novel, promising avenue for Alzheimer’s disease drug development.

The Lee et al. paper represents one of the earliest large-scale studies comparing mutations in normal and diseased tissues, and it already generated promising insights about mechanism, diagnosis, and potential therapy [47]. Another paper published around the same time performed comprehensive single nucleotide variant (SNV) identification using single-cell whole-genome sequencing [50]. The investigators took 161 neurons from the prefrontal cortex and hippocampus of fifteen healthy individuals of all ages as well as nine individuals who had early-onset neurodegeneration due to the genetic disorders of Cockayne syndrome and Xeroderma pigmentosum. While SNVs increased linearly in both cohorts, they were more common in the individuals with neurodegenerative disease. A higher rate of mutation was also observed in the hippocampus, and the authors posit that this may be relevant for Alzheimer’s disease.

Altogether, the investigators delineated three mutational signatures [50]. The first signature, characterized by C>T and T>C mutations, increased with age, in a clock-like fashion. Perhaps related to early development, the second signature featured primarily C>T mutations and did not change with age. Finally, the third signature had C>A variants and was connected with oxidative DNA damage.

Some research has also been conducted surrounding aneuploidy (chromosome copy number variations) in the brain which occurs as a result of errors in mitosis [51]. While aneuploidy is highly common in cancer, surprisingly high levels of aneuploidy have also been observed in the normal brain [52, 53]. As expected, its frequency increases with age though the brain is still able to function normally despite elevated amounts of mosaic aneuploidy [54, 55]. At abnormally high levels, aneuploidy can lead to significant declines in neural function, including Alzheimer’s and Parkinson’s disease [56, 57].

While intriguing, this complex field is still in its infancy and the connections between neural mosaicism and normal function or neurodegeneration are unclear. More research is needed in order to conclusively determine what genetic changes are not cause for concern or those that are associated with devastating conditions including Alzheimer’s disease and Cockayne syndrome.

## Conclusions

Studying the transition of tissues from normal to diseased may enable early diagnosis as well as mechanisms for targeted pharmacology. Even though this branch of science is in its infancy, the scientific dogma is changing with the awareness that heavily mutated tissues are not necessarily hallmarks of disease but are actually the new normal [58]. There is greater awareness that an exclusive focus on studying tumor or diseased tissue limits our ability to learn about precancerous or pre-disease states. Moreover, advances in sequencing techniques and bioinformatics may uncover more insights into this rapidly developing field, such as the prevalence of positive versus negative selection in cancer development [59]. Consequently, more studies and projects are addressing this dearth of information, studying genetic events and clonal properties from a wide variety of tissue samples and participants [21].

One such initiative is the National Cancer Institute’s Pre-Cancer Genome Atlas [60], a large-scale collaboration to create a comprehensive database of histological and genomic/proteomic data from premalignant lesions which will produce new opportunities for early detection and risk stratification. Advances in nucleic acid sequencing including massively parallel as well as methylation-based techniques will also be helpful to accurately detect mutated or altered DNA in both normal and diseased tissues. Conversely, a new approach employing patterns in fragment length to distinguish cfDNA from ctDNA has also yielded some promising results for early cancer diagnosis though this method may have important limitations [61–63].

It is clear that it is unfeasible to diagnose cancer simply by examining mutations. Some recent studies go beyond mutations to consider chromatin organization. One recent study by Liu et al. studied DNA methylation as a further hallmark of a cancer cell, beyond simply mutation [64]. Another major investigation combined the detection of oncogenic ctDNA mutations, protein markers, and PET-CT (positron emission tomography—computerized tomography) in order to increase the accuracy of cancer detection while specifying the tissue of origin [65].

Other advances may come from creatively utilizing existing databases. For example, Garcia-Nieto et al. employed RNA-sequencing data to study mutagenesis in 7500 healthy tissue samples, coming from 36 distinct tissues [66]. Through examining the transcriptome, they were able to delineate somatic tissue evolution through examining the positive and negative selection for various mutations. Apart from some common cancer mutations that were highly enriched (like missense mutations in IDH2 or excess synonymous mutations in MP2K1), the team found strong negative selection acting on the majority missense and nonsense mutations.

A collection of articles published in Nature in May 2020 by the Genome Aggregation Database (gnomAD) Consortium constitutes another major step forward. Karczewski et al. examined predicted loss of function (pLoF) mutations using whole-genome sequencing (WGS) and exome sequencing of a diverse population of 141,456 individuals [67]. By comparing the expected and actual frequency of pLoF variants, the authors were able to classify the genes based on pLoF tolerance (from no effect to lethal). Another paper in the collection examined differences in tolerance of genes to pLoF, demonstrating that many of these variants appear in exons which minimize their effect [68]. The other papers discussed the potential utility of the gnomAD database in drug discovery [69] and conducted a large-scale analysis of structural variants in protein coding and non-coding regions [70].

Investigating mutations in normal tissues requires enormous amounts of data, particularly as these changes can be rare and subtle. It is particularly important to include genetic data from individuals from underrepresented racial and ethnic backgrounds as well as people of all ages. Continued innovations stemming from big data and precision medicine will be highly valuable; they will enable very high-resolution studies of normal tissues and diseased tissues as well as new biomarker discovery to facilitate better outcomes for individuals with cancer, Alzheimer’s disease, cardiac pathologies and beyond.

## Data Availability

Not applicable

## Abbreviations

AML: Acute myeloid leukemia
APP: Amyloid-β precursor protein
ARCH-PD: Age-related hematopoiesis putative driver mutation
ctDNA: Circulating tumor DNA
cfDNA: Cell-free DNA
gencDNAs: Genomic complementary DNA
PET-CT: Positron emission tomography—computerized tomography
